# Supplementary material for: Cajanin Suppresses Melanin Synthesis through Modulating MITF in Human Melanin-Producing Cells
Source: Molecules. 2021 Oct 5;26(19):6040. doi: 10.3390/molecules26196040 (PMC8512678; doi:10.3390/molecules26196040)
Supplement: Supplementary file 1 [file molecules-26-06040-s001.zip › molecules-1336499-supplementary.pdf]

molecules

Supplementary Materials

Cajanin Suppresses Melanin Synthesis through Modulating MITF in Human Melanin-Producing Cells

Ponsawan Netcharoensirisuk [1], Kaoru Umehara [2], Wanchai De-Eknamkul [3,*] and Chatchai Chaotham [1,4,*]

[1] Department of Biochemistry and Microbiology, Faculty of Pharmaceutical Sciences, Chulalongkorn University, Bangkok 10330, Thailand; Ponsawan.Ne@student.chula.ac.th
[2] Faculty of Pharmaceutical Sciences, Yokohama University of Pharmacy, Kanagawa 2450066, Japan; kaoru.umehara@hamayaku.ac.jp
[3] Department of Pharmacognosy and Pharmaceutical Botany, Faculty of Pharmaceutical Sciences, Chulalongkorn University, Bangkok 10330, Thailand
[4] Cell-Based Drug and Health Products Development Research Unit, Faculty of Pharmaceutical Sciences, Chulalongkorn University, Bangkok 10330, Thailand
* Correspondence: Wanchai.D@chula.ac.th (W.D.-E.); cchoatham@gmail.com (C.C.)

Table of Content

Figure S1. Growth curve of MNT1 cells

Figure S2. The melanin production in MNT1 cells after culture for 0–24 h.

Figure S3. Tyrosinase activity in MNT1 cells cultured for 0–12 h.

Figure S4. The mRNA expression levels of melanogenesis-related proteins in human MNT1 cells cultured with cajanin for 0–12 h.

Figure S5. The expression levels of melanogenesis-related proteins in human MNT1 cells cultured with cajanin for 0–12 h.

Figure S6. The expression levels of signaling molecules mediating MITF expression in human MNT1 cells cultured with cajanin for 0–12 h.

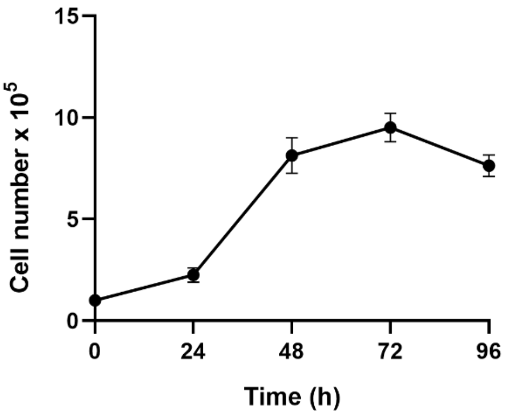

Figure S1. Growth curve of MNT1 cells. Human melanoma MNT1 cells were seed at density of 1 × 10^5 cells/well into 6-well plates and further cultured for 24–96 h. The number of cells at each time point were counted after staining with tryptan blue and generated the growth curve. Based on analysis via GraphPad Prism 9.0 software, the MNT1 cells under the present condition possess the doubling time at 13.75 h. Experiments were performed in triplicate and data are expressed as mean ± SEM.

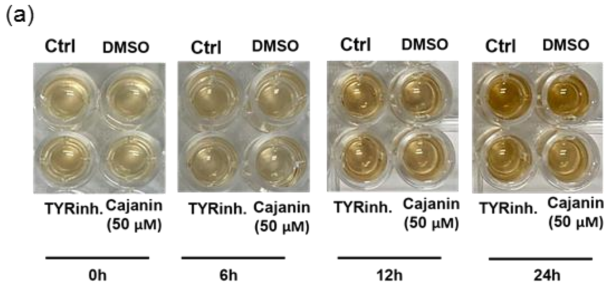

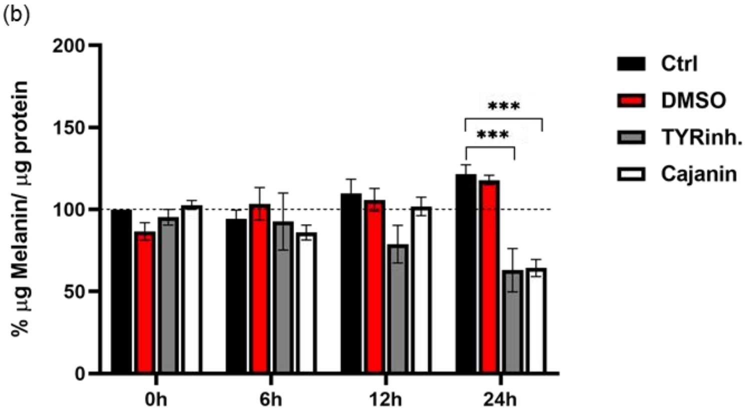

Figure S2. The melanin production in MNT1 cells after culture for 0-24 h. (**a**) Treatment for 0–12 h with neither 50 μM cajanin nor 20 μM tyrosinase inhibitor (TYRi; 4-butylresorcinol) altered melanin production in human MNT1 cells. (**b**) During the incubation period of 0-24 h, the gradual increase of melanin content was early noted in human melanin-producing MNT1 cells since 12 h. DMSO was used as a vehicle control. Experiments were performed in triplicate and data are expressed as mean ± SEM. *** $p < 0.005$ versus non-treated cells (Ctrl).

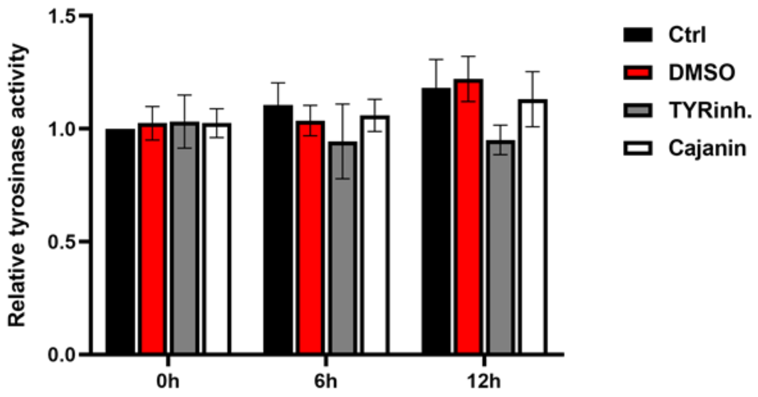

Figure S3. Tyrosinase activity in MNT1 cells cultured for 0-12 h. Human MNT1 cells at density of 1 × 10^5 cells/well in 6-well plate were treated with 50 μM cajanin, 20 μM tyrosinase inhibitor (TYRi; 4-butylresorcinol) or DMSO (vehicle) for 0-12 h before being subjected for determining tyrosinase activity via cell-based assay. There was no significant alteration of tyrosinase activity in all treatment groups compared with the non-treated control cells at the same time point. Additionally, the minor increase of tyrosinase activity relatively to 0 h was observed in MNT1 cells cultured for 6-12 h. Experiments were performed in triplicate and data are expressed as mean ± SEM.

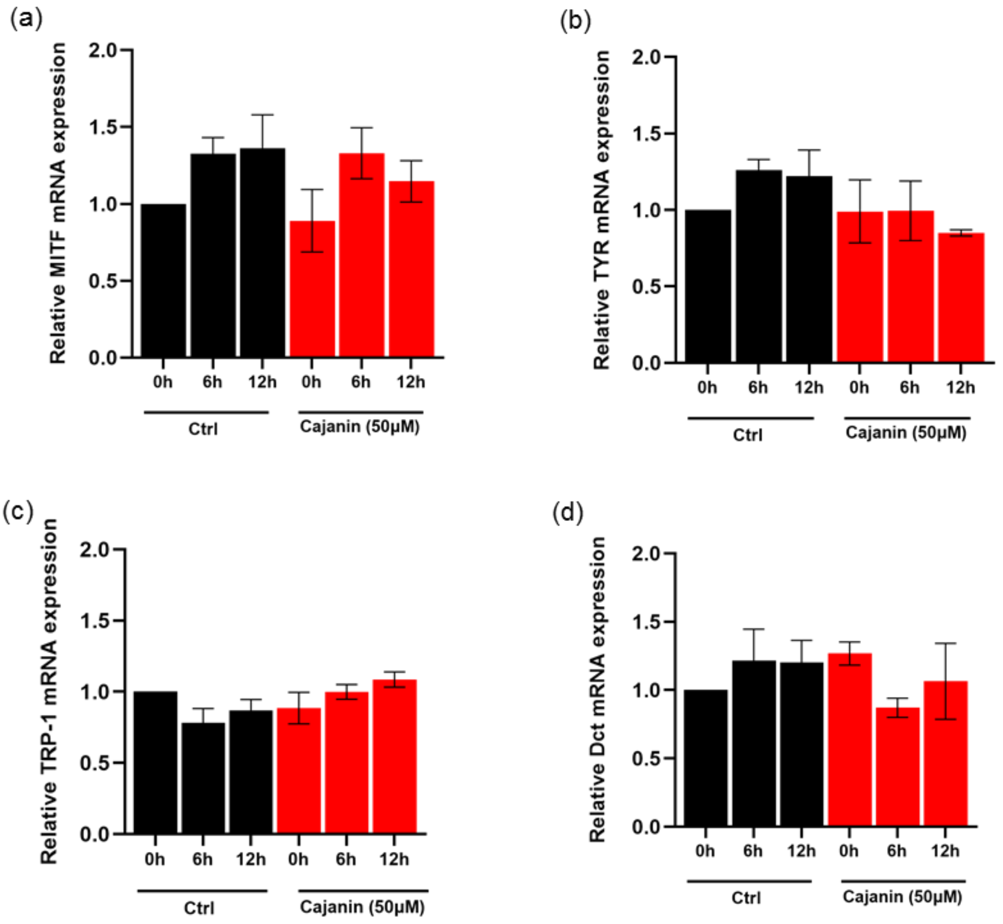

Figure S4. The mRNA expression levels of melanogenesis-related proteins in human MNT1 cells cultured with cajanin for 0–12 h. Real-time quantitative RT-PCR revealed no significant alteration of mRNA levels of (**a**) MITF, (**b**) tyrosinase (TYR), (**c**) TRP-1 and (**d**) Dct in MNT1 cells cultured with 50 μM cajanin for 0–12 h compared with non-treated control cells at the same time point. Although minor alteration of the mRNA levels was observed in MNT1 cells cultured for 6–12 h, there was not significantly different when compared with the cells at 0 h. Data obtained from qRT-PCR were normalized to GAPDH expression level and represented relative to control at 0 h. Experiments were performed in triplicate and data are expressed as mean ± SEM.

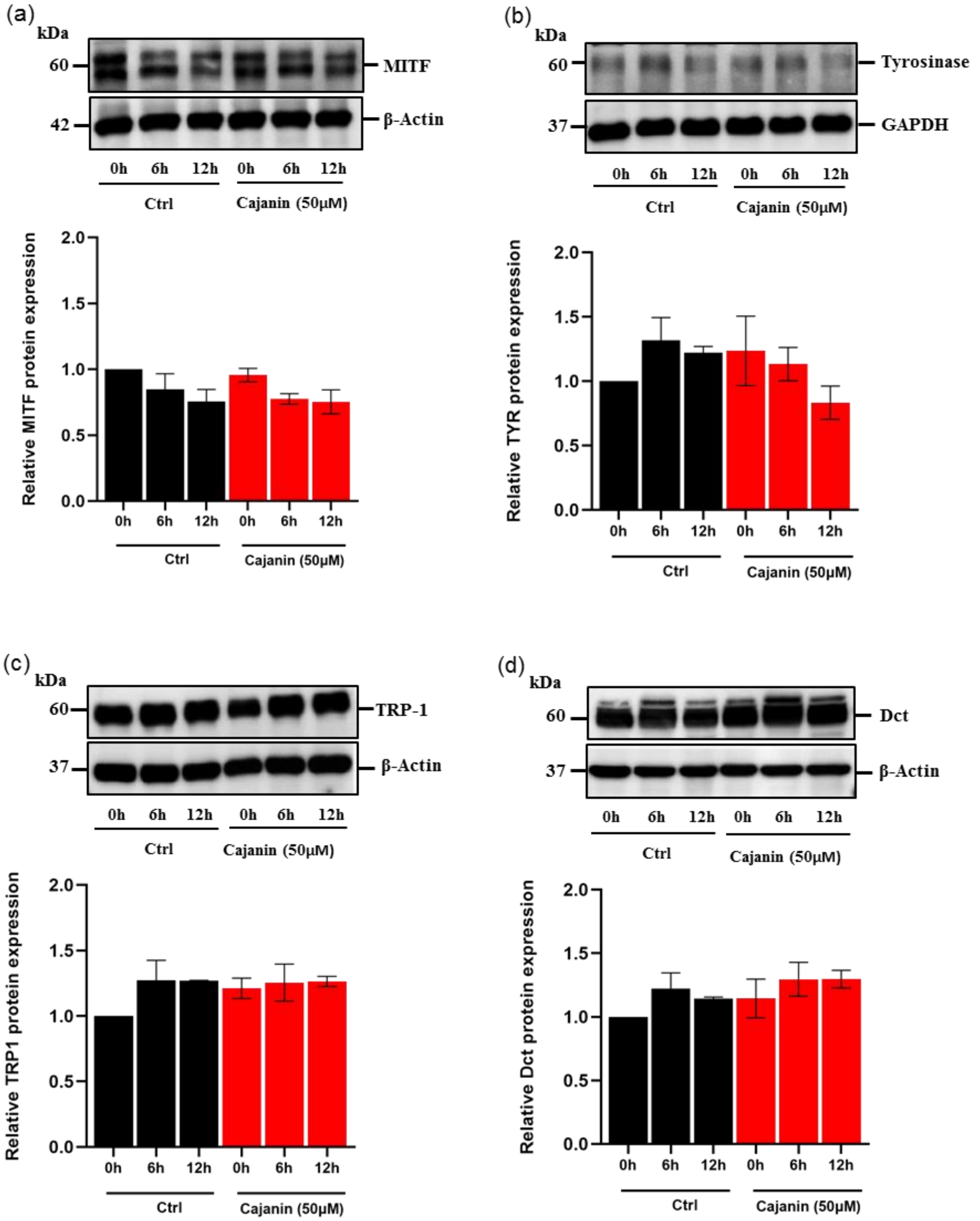

Figure S5. The expression levels of melanogenesis-related proteins in human MNT1 cells cultured with cajanin for 0–12 h. Western blot analysis demonstrated that there was no significant alteration of (**a**) MITF, (**b**) tyrosinase (TYR), (**c**) TRP-1 and (**d**) Dct protein levels in MNT1 cells treated with 50 μM of cajanin for 0–12 h compared with non-treated control cells. Experiments were performed in triplicate and data are expressed as mean ± SEM.

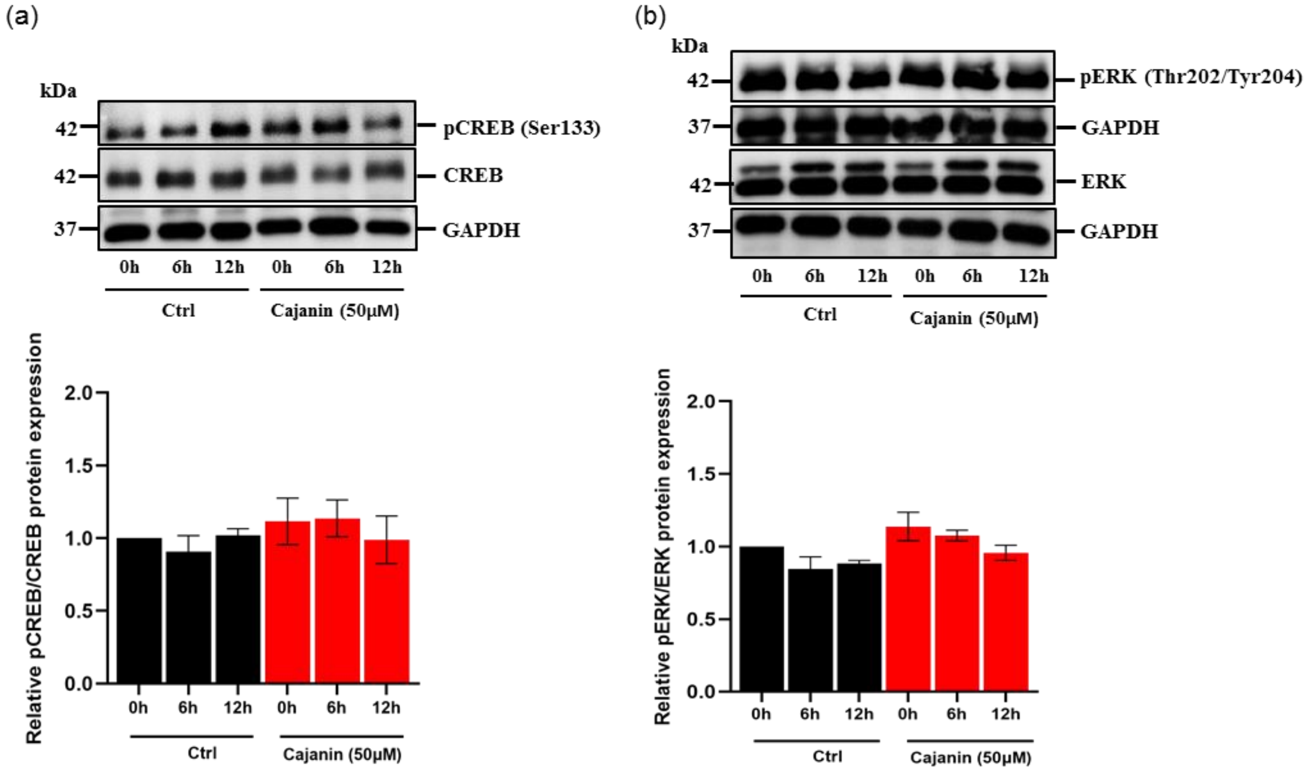

Figure S6. The expression levels of signaling molecules mediating MITF expression in human MNT1 cells cultured with cajanin for 0–12 h. Western blot analysis demonstrated that there was no significant alteration of (**a**) pCREB/CREB and (**b**) pERK/ERK levels in MNT1 cells treated with 50 μM of cajanin for 0–12 h compared with non-treated control cells. Experiments were performed in triplicate and data are expressed as mean ± SEM.
